# The Implementation of X + Y Scheduling in Combined Internal Medicine-Pediatrics Residency Programs: Practical Considerations for Program Leadership

**DOI:** 10.7759/cureus.29743

**Published:** 2022-09-29

**Authors:** Nathan R Stehouwer, Michael R Contarino, Dava Szalda, Kathryn Diamond-Falk, Jennifer B Walsh

**Affiliations:** 1 Internal Medicine-Pediatrics, University Hospitals Cleveland Medical Center and Rainbow Babies & Children's Hospital, Cleveland, USA; 2 Internal Medicine-Pediatrics, University of North Carolina Health, Chapel Hill, USA; 3 Internal Medicine-Pediatrics, Children's Hospital of Philadelphia and Hospital of the University of Pennsylvania, Philadelphia, USA; 4 Internal Medicine-Pediatrics, Maine Medical Center, Portland, USA; 5 Internal Medicine-Pediatrics, University of Texas Southwestern Medical Center, Dallas, USA

**Keywords:** internal medicine-pediatrics, x + y scheduling, ambulatory education, continuity clinic, med-peds residency, block scheduling

## Abstract

The X + Y scheduling approach, or block scheduling, is common among internal medicine residency programs. With the beginning of a pilot program through the American College of Graduate Medical Education in 2018, pediatrics and internal medicine-pediatrics (Med-Peds) residency programs have been able to adopt X + Y scheduling as well. The X + Y scheduling approach presents unique challenges and opportunities for combined Med-Peds residencies. This paper describes an early experience with X + Y scheduling in Med-Peds residencies and describes practical considerations for Med-Peds programs considering or planning a transition to the X + Y schedule. These considerations include strategies for gaining stakeholder support; selecting the appropriate block structure; opportunities for designing the ambulatory curriculum; and maximizing the clinical benefit in the residency continuity clinic.

## Editorial

Introduction

Over the last decade, X + Y scheduling models have gained popularity among internal medicine residency programs [1-3]. X + Y scheduling, or block scheduling, refers to a system in which residents alternate between blocks of traditional resident rotations and blocks of ambulatory, primary care-focused experiences. X refers to the number of weeks spent per block of traditional rotations, which may include wards, intensive care units, electives, emergency departments, and vacations. Y refers to the number of weeks spent in ambulatory blocks, which include immersion in continuity clinics as well as other ambulatory-focused experiences. Typically, X and Y blocks alternate in a regular cycle.

Recently, residency programs in pediatrics gained the flexibility to pilot X + Y models with the advent of a pilot program in 2018 available through the American College of Graduate Medical Education (ACGME), the Advancing Innovation in Residency Education (AIRE) program. This program granted an exception to the requirement that continuity clinics be distributed over 26 weeks per year in pediatrics and combined internal medicine-pediatrics (Med-Peds) residencies, allowing flexibility for training programs seeking to enact block scheduling. Previously, most Med-Peds programs utilized a traditional format in which weekly half-day clinics were distributed throughout inpatient and ambulatory rotations, though some programs utilized an X + Y format with modifications to maintain compliance with the ACGME 26-week requirement. The 2021 Med-Peds Program Directors Survey showed that 38% of Med-Peds programs had at least one categorical program using X + Y scheduling, and 22% of responding programs were participating in X + Y scheduling models in both categorical programs. The authors of this study are program directors of five of the first Med-Peds programs to adopt X + Y scheduling under the AIRE pilot; in this article, we aim to use our programs’ experience to describe unique considerations for Med-Peds programs and provide a guide for Med-Peds program directors considering X + Y scheduling. In so doing, we will supplement the work of others by describing more general considerations of X + Y implementation for categorical residency programs [1-3]. In this paper, we divide considerations for a Med-Peds program considering or planning an X + Y curriculum into four areas, each of which has unique challenges and opportunities for combined training programs (Table 1).

**Table 1:** Considerations for combined internal medicine-pediatrics residency programs planning X + Y curriculum

Task	Key considerations
Gaining stakeholder support	Internal medicine and pediatrics leadership
	Rotation directors
	Clinic faculty
	Residents
	Institutional leadership
	Patients
Selecting the appropriate block structure	Match categorical schedules where possible
	Consider inpatient service needs in categorical programs
	Consider continuity clinic service need
	Re-evaluate the educational value of rotations and consider how many rotations can be reduced or incorporated into Y-blocks
	Consider the value of flexibility and modular structures
	Consider optimal spacing and density of clinics to support continuity
	Consider whether Y-blocks will be used for immersion rotations along with continuity clinics
	Plan for rotations which will change from four weeks to two-week blocks
Redesigning the ambulatory curriculum	Consider which rotations can be included in ambulatory blocks (such as adolescent medicine, advocacy, neurology, and geriatrics)
	Consider which non-continuity ambulatory rotations will be incorporated into ambulatory blocks longitudinally or by immersion
	Consider the use of pre-clinical or academic half-day didactic curricula, both of which can be facilitated by X + Y scheduling
	Plan for the use of resident cohorts for advising, wellness activities, and team building
Maximizing the benefit for continuity clinic	Plan resident team structure to enhance continuity
	Ensure advance scheduling of resident clinics
	Ensure adequate resident staffing
	Plan for clinic and ambulatory block timing to avoid long gaps between resident continuity clinics

Gaining stakeholder support

Combined Med-Peds programs should address the priorities of a broad range of stakeholders as they consider the X + Y schedule. Med-Peds program directors need support from institutional leadership and program faculty but also must navigate the implications of X + Y scheduling for their partners in internal medicine and pediatrics. In particular, where scheduling alignment with categorical programs is desired, gaining support from categorical internal medicine (IM) and pediatrics program leadership is important.

IM programs have had longer experience with X + Y curricula, with results that can help Med-Peds program directors build the case for converting to X + Y scheduling. Studies of IM residency programs and block scheduling have demonstrated consistent benefits of decreased educational fragmentation [4], increased resident satisfaction [5], and decreased resident stress. From a curricular perspective, IM programs have reported greater flexibility to implement longitudinal curricula and increase ambulatory training with X + Y scheduling. Importantly, 90% of IM program directors who use X + Y scheduling report satisfaction with having made the change to X + Y [6]. Program directors may find they have additional time and structure for additional curricular priorities such as quality improvement [7].

A decade of experience in internal medicine residency can make a compelling case for X + Y scheduling for pediatric residency program leaders. Additionally, emerging literature specific to pediatrics programs may be helpful. Many pediatrics leaders and regulators have historically had concerns about the impact of X + Y scheduling on patient continuity in outpatient clinics, which may have different implications in pediatrics than in IM outpatient clinics. However, Osborn [8] showed overall improvement in continuity after implementation of an X + Y curriculum, and Myers [9] reported substantially increased patient continuity based on resident self-report, findings consistent with those reported by internal medicine residencies [5]. Pediatric programs may also be encouraged given the reports of increased teaching time from the AIRE pilot [9]. Multiple models exist for implementing X + Y curricula, and like many educational interventions, the benefits are likely to depend on the matching of implementation strategies to local resources and needs.

Program faculty need to be included early in planning for a transition to X + Y. Inpatient and ICU faculty should be informed of the positive benefits of providing a consistent day-to-day resident team. For outpatient clinic faculty, the opportunities presented by a group of residents who are more engaged, focused, and energetic without the competing demands of high-acuity inpatient care can be emphasized. Faculty who oversee rotations that may be substantially impacted must be included early in the planning phases. For example, many X + Y structures that require at least four-week rotations may be converted into two-week rotations, and rotations may be converted from immersion to longitudinal formats. The details of these strategies are discussed below. Faculty involved in highly affected rotations may have concerns about potential negative experiences, and their concerns should be addressed early. All rotation directors should be included early to strategize for optimal implementation.

In our experience, Med-Peds residents are enthusiastic adopters of the change to X + Y scheduling. At one of our programs, which tracked annual burnout rates among residents starting before the adoption of X + Y scheduling, several years of sustained improvements in burnout rates were observed following conversion to X + Y scheduling. To enhance resident support for a change to X + Y, it may be helpful to facilitate conversations with residents at programs with X + Y scheduling to directly exchange experiences and ideas.

Support from institutional leadership and program chairs can be gained by emphasizing improved trainee wellness and satisfaction, decreased potential for ACGME duty hours violations, and the possibility of fewer safety events given decreases in handoffs and distractions [9].

Selecting the appropriate block structure

Multiple X+Y models exist. The most common models are 4+1 and 6+2, but 4+2 and 3+1 are also common. Some internal medicine programs have also used 4+4, 5+1, and 8+2, or even variable formats. The right model for each Med-Peds residency depends on several factors.

**Figure 1:**
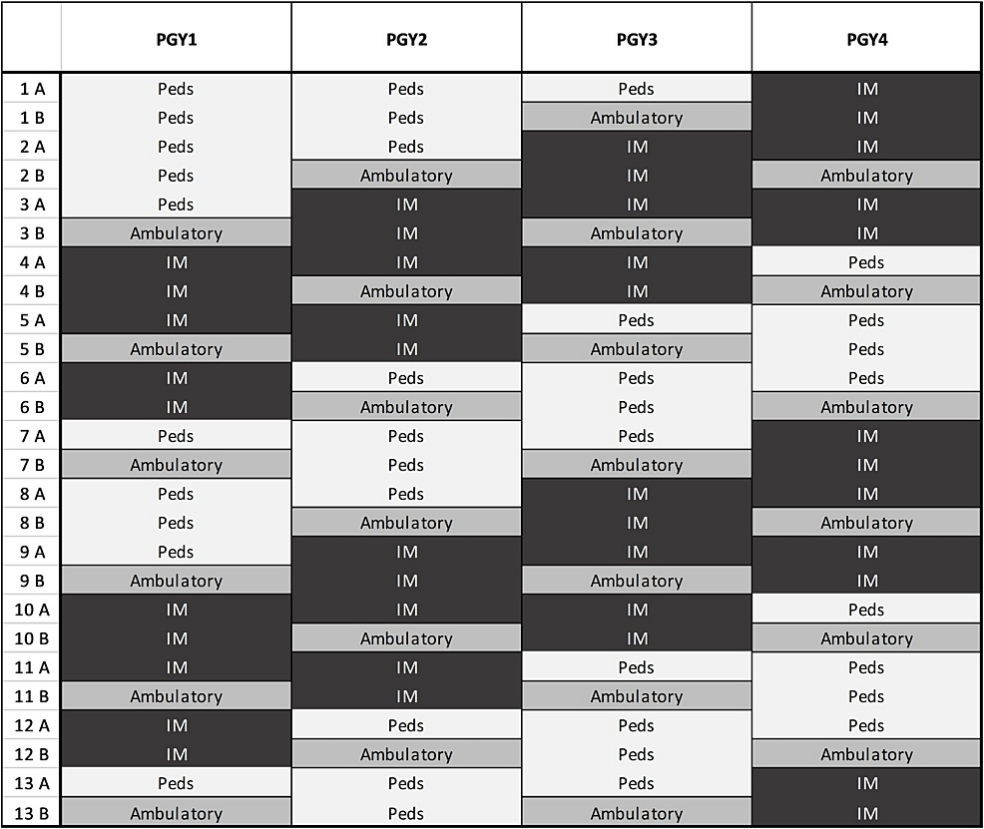
Matched 6 (X) + 2 (Y) schedules This is a sample schedule for a single resident; half of the residents in this program would follow a mirror image of this schedule so that half of the Med-Peds residents are distributed to each categorized program at the same time. All ambulatory blocks include both pediatric and adult continuity clinic experiences. Blocks marked IM and Peds reflect traditional X-block rotations. All blocks are two weeks in duration.

**Figure 2:**
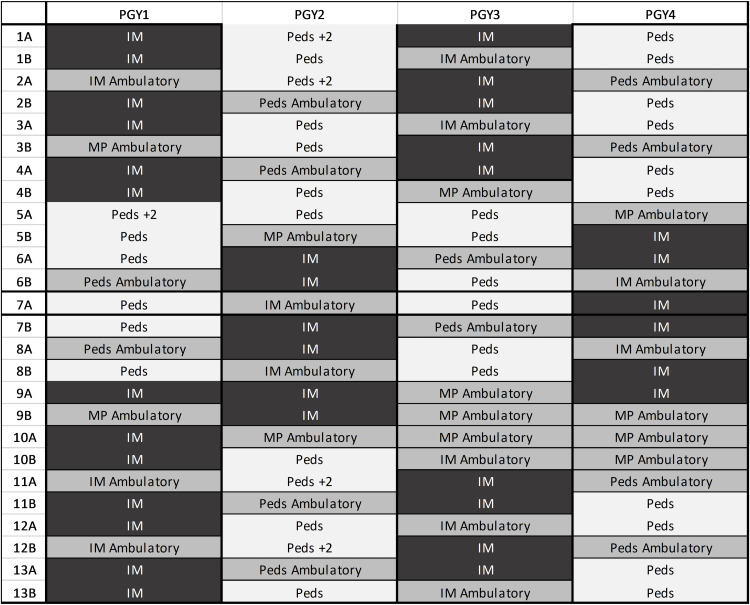
Med-Peds scheduling for discordant X + Y (6 + 2 and 4 + 1) Blocks marked IM and Peds reflect traditional X-block rotations. To optimally align IM-Peds switch days, the length of blocks is not always consistent, but a total of 26 weeks in each categorical program per year is maintained. Two-week pediatric rotations are incorporated strategically to balance time between categorical programs.

**Figure 3:**
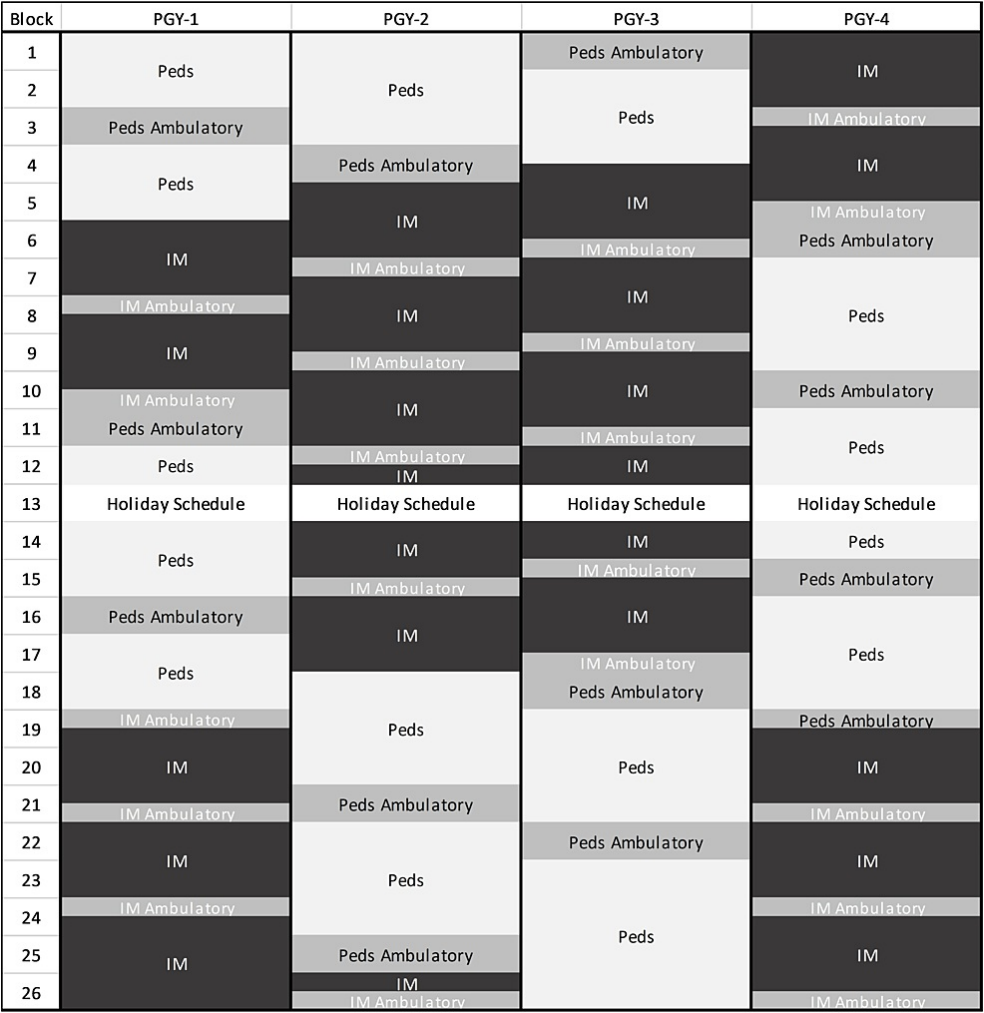
4 (X) + 2 (Y) scheduling at a Med-Peds program with only IM categorical program using X + Y Some pediatrics blocks did not align with an ambulatory block. In these situations, residents would have a continuity clinic for one to two half-days, similar to the traditional schedule, and have an extra clinic during an ambulatory block elsewhere. These Peds blocks, which include continuity clinic times but are otherwise not full ambulatory blocks, are marked as Peds+2. Ambulatory blocks can be designated as Peds, IM, or Med-Peds (MP), but all include a Med-Peds continuity clinic. Blocks marked IM and Peds reflect traditional X-block rotations.

The first decision point for a Med-Peds program director is based on the existing scheduling structures in the participating internal medicine and pediatrics programs. Matching existing X + Y schedules in internal medicine and pediatrics allows for smoother rotation transitions, simpler scheduling, and greater flexibility for Med-Peds residents to utilize categorical ambulatory didactic and clinical experiences. Therefore, we recommend that, where possible, Med-Peds leadership advocate for their categorical counterparts to adopt matching X + Y structures. However, even when internal medicine and pediatrics programs have non-matching X + Y structures, X + Y scheduling can be accomplished by Med-Peds residencies. Med-Peds residencies can also implement X + Y when only one categorical program is using X + Y scheduling. Each of these situations is represented in the authors’ residency programs, and we provide example templates (Figures 1-3).

Med-Peds programs must then consider the total proportion of time which can be allocated to ambulatory Y-blocks; this can range from 20% or less (4+1 or 8+2 models) to 50% (4+4 models). The size of programs’ demand for inpatient service may constrain choices; additionally, for curricular reasons, Med-Peds program directors may choose a format with a higher percentage of time in traditional rotations for curricular reasons. The need for clinic staffing may necessitate a minimum frequency of ambulatory blocks. Implementation of X + Y scheduling will require decreasing the number of blocks allocated to traditional residency rotations to accommodate the Y curricular time. This can be accomplished by eliminating some rotations and incorporating others into ambulatory blocks, a concept that is discussed further below. Compared with ACGME requirements and program aims, existing block schedules may highlight rotations that can be shortened or eliminated. A shift to X + Y scheduling may offer an opportunity for Med-Peds programs to adjust the degree of Med-Peds participation in various categorical rotations to maximize their educational exposure and optimize the balance of inpatient and outpatient training.

The flexibility of scheduling may be a consideration for programs, particularly for Med-Peds residencies, which already have less scheduling flexibility than their categorical counterparts. More modular designs, such as 6 + 2 or similar variations, in which ambulatory occupies the same rotation slot as any other rotation, may allow for greater scheduling flexibility, a greater ability for residents to switch rotations, and an easier time dealing with resident absences, etc.

Optimal spacing for patient continuity should be considered. Local factors dependent on each program’s patient population and clinic structure may impact the optimal spacing for that program. The spacing of clinics for optimal continuity may differ between pediatric and adult patients. The Med-Peds programs continue to have different ACGME requirements for several patient encounters for adult and pediatric patients that need to be tracked that differ from the categorical programs [10].

In general, more frequent ambulatory blocks (such as in 3+1 or 4+1) may have advantages for resident continuity and experience in the clinic. However, this question has not yet been studied, and a high density of clinic availability on a bimonthly basis as in 6+2 or 4+4 structures may be beneficial as this structure supports the typical spacing of well-child care.

Programs should consider whether non-continuity ambulatory experiences will be incorporated as longitudinal experiences or immersion as discussed below. If the answer is by immersion, two-week ambulatory blocks may be preferable to one-week blocks to allow for a robust experience.

Other implications of X + Y scheduling (such as planning for rotation handovers occurring at the same time for all residents or on a rolling basis) are important but are not unique to Med-Peds, and are discussed elsewhere in the literature [1].

Redesigning the ambulatory curriculum

Conversion to X + Y scheduling offers a substantial opportunity for innovation and improvement of the ambulatory curriculum. Med-Peds programs must determine how to draw on ambulatory opportunities in both internal medicine and pediatrics and when to insert programming unique to Med-Peds residents to meet ambulatory training goals and objectives. Our programs have found that many educational experiences are well-suited to incorporation into ambulatory Y-blocks. Primary care-focused didactics can be integrated with categorical ambulatory didactics. Clinical experiences which augment primary care training, such as sports medicine, neurology, geriatrics, women’s health, and clinical panel management, can be incorporated on a planned and longitudinal basis to capitalize on the benefits of distributed learning. Depending on local constraints and resources, many required rotations can be considered for integration into Y-blocks, including child advocacy, community pediatrics, developmental and behavioral pediatrics, adolescent medicine, and quality improvement.

**Figure 4:**
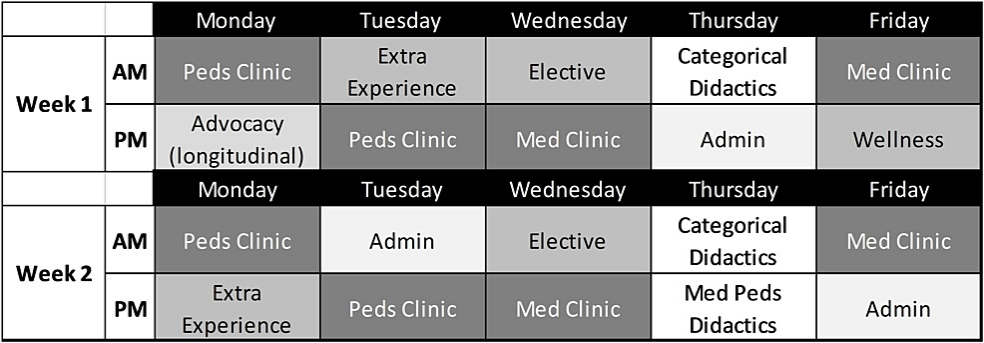
An example of an ambulatory Y-block from a Med-Peds program which utilizes separate IM and Pediatrics clinics

Residencies converting to X + Y scheduling have utilized ambulatory blocks for both immersion experiences as well as longitudinal distributed learning approaches where a given experience is repeated over multiple blocks. For example, a given Y-block may emphasize neurology or geriatrics to meet ACGME requirements. Longitudinal experiences may be particularly beneficial for career development and mentorship as residents may develop longitudinal relationships with faculty in various areas, not only in their primary care clinic. Programs need to plan to ensure that experiences which are implemented longitudinally or modified into immersion block structures still meet ACGME definitions for time blocks after accounting for time spent in the continuity clinic. An example Med-Peds Y block structure can be seen in Figure 4.

There are several ways to incorporate didactic ambulatory education into the X+Y model. The guaranteed rotation of cohorts through the Y-block can enhance the ability to plan a curriculum that will be experienced by all residents. Pre-clinic conferences and ambulatory-focused resident case presentations can be incorporated just as in traditional scheduling models. With X + Y scheduling, programs also have the option of creating didactic sessions for each cohort that may include IM ambulatory didactics, pediatric ambulatory didactics, Med-Peds specific ambulatory didactics, or a mixture of all three.

In addition to clinical education, the grouping of residents into Y-block cohorts can be used for advising, resident wellness, and team building. In Med-Peds, a cohort may be made up of a specific class, which allows more class bonding time. A cohort may also be composed of a specific mentoring group. Social and evening activities can also be more easily scheduled during the Y-block time.

Med-Peds programs that utilize a combined Med-Peds clinic will need to ensure adequate staffing during all ambulatory blocks; programs that utilize separate IM and pediatrics clinics will need to coordinate with categorical programs to ensure adequate staffing if Med-Peds residents are not evenly distributed across Y blocks, or if the IM and pediatrics programs utilize different X + Y schedules.

Maximizing the benefits of continuity clinic

X + Y scheduling has the potential to improve patient continuity [9], but to capitalize on this opportunity, Med-Peds programs and clinics must plan for adequate clinic staffing. Ensuring adequate clinic staffing can require extra planning in Med-Peds programs, whose residents are typically distributed among rotations in two categorical residency programs.

Patient continuity is a frequent challenge for residency clinics with or without block scheduling. X + Y scheduling presents opportunities to improve patient continuity. Strategies to develop continuity include ensuring the provider schedule is set at least three months ahead of time, and ideally six months ahead. This enables routine one, two, and three-month follow-ups to be scheduled with the same provider, especially for well-child checks during the first year of life. We have found that, because of the template nature of block scheduling, advanced scheduling of resident clinics is facilitated by X + Y. Clinics can create a waitlist for follow-up appointments when the provider is not yet scheduled. Attention should be paid to ensuring that vacations, holiday schedules, transitions between IM and peds, and year-to-year transitions do not result in long gaps between ambulatory blocks, which could disrupt continuity. Lastly, programs must ensure that residents have large enough patient panels to fill clinic appointments regularly to allow resident physicians to see a high percentage of continuity patients during clinic days.

Creating primary care teams can further augment patient continuity and panel management. In most X + Y systems, the residency will be divided into cohorts that rotate sequentially through Y-blocks. Teams should be composed of 1-2 residents from each cohort, creating a small group practice within the resident clinic where they share patient panels and respond to urgent requests and questions while the primary resident is not on ambulatory rotations. The sense of a small team responsible for patient care may enhance both patient and resident perceptions of continuity and improve resident training in panel management. The time afforded to ambulatory experience in block scheduling models may also be used to more explicitly teach and promote panel management skills. Creating a coverage schedule to best support schedule demands and enable accountability between residents has the best chance for success. Resident buy-in is essential for any patient coverage scheme, which should also include clinic staff visibility for communication purposes.

Programs with separate internal medicine and pediatric clinics can split time between the clinics to minimize long gaps of time away from either specialty. Full days or half days at each clinic may be preferable depending on travel time and other scheduling factors.

Conclusion

The development of X + Y scheduling represents an exciting opportunity for Med-Peds programs to innovate and improve multiple aspects of their programs, including patient continuity, inpatient workflow, inpatient and outpatient education, and resident wellness. Implementation of X + Y scheduling involves many decisions, which present unique considerations for combined Med-Peds program leadership. Future research will examine the implications of these decisions from the perspectives of Med-Peds residents to better inform future scheduling models.
